# Telomere dysfunction suppresses multiple endocrine neoplasia in mice

**DOI:** 10.18632/genesandcancer.31

**Published:** 2014-09

**Authors:** Ji-Hyeon Lee, Miriam Anver, Maria Kost-Alimova, Alexei Protopopov, Ronald A. DePinho, Sushil G. Rane

**Affiliations:** ^1^ Diabetes, Endocrinology & Obesity Branch, National Institute of Diabetes & Digestive & Kidney Diseases, Bethesda, MD; ^2^ Leidos Biomedical Research, Inc., Frederick National Laboratory for Cancer Research, Frederick, MD; ^3^ Dana-Farber Cancer Institute, Boston, MA; ^4^ Department of Genomic Medicine, University of Texas MD Anderson Cancer Center, Houston, TX; ^5^ Department of Cell Biology, University of Texas MD Anderson Cancer Center, Houston, TX

**Keywords:** Telomerase, Pituitary, Pancreas, Islets, Endocrine Neoplasia

## Abstract

Multiple endocrine neoplasia (MEN) syndrome is typified by the occurrence of tumors in two or more hormonal tissues. Whereas the genetics of MEN syndrome is relatively well understood, the tumorigenic mechanisms for these cancers remain relatively obscure. The *Cdk4*^R24C^ mouse model develops highly penetrant pituitary tumors and endocrine pancreas adenomas, and, as such, this model is appropriate to gain insight into mechanisms underlying MEN. Using this model, here we provide evidence supporting an important role for telomerase in the pathogenesis of MEN. We observed increased aneuploidy in *Cdk4*^R/R^ fibroblasts along with significantly elevated telomerase activity and telomere length in *Cdk4*^R/R^ islets and embryonic fibroblasts. To better understand the role of telomerase, we generated *Cdk4*^R24C^ mice with inactivation of the *mTERC* locus, which codes for the essential RNA component of the enzyme telomerase (*mTERC*^−/−^ *Cdk4*^R/R^ mice). Embryonic fibroblasts and islets derived from *mTERC*^−/−^ *Cdk4*^R/R^ mice exhibit reduced telomere length and proliferative capacity. Further, *mTERC*^−/−^ *Cdk4*^R/R^ fibroblasts display reduced transformation potential. Importantly, *mTERC*^−/−^ *Cdk4*^R/R^ mice display significantly reduced spontaneous tumorigenesis. Strikingly, we observed dramatic suppression of pituitary tumors and endocrine pancreas adenomas in *mTERC*^−/−^ *Cdk4*^R/R^ mice. Telomere dysfunction suppressed tumor initiation and increased latency of tumor development while not affecting the progression of established tumors. In summary, these results are suggestive of an important role for telomerase in tumor development in the *Cdk4*^R24C^ mouse model, specifically in the genesis of tumors in the pituitary and the endocrine pancreas.

## INTRODUCTION

Multiple endocrine neoplasia (MEN) is clinically defined as a disorder with tumors arising in two or more different hormonal tissues [1, 2]. MEN1 is an autosomal dominant cancer syndrome that is characterized by multiple tumors in the endocrine pancreas, the anterior pituitary and the parathyroid glands [1]. Inactivation of the *MEN1* locus, that encodes the tumor suppressor protein ‘menin’, is the hallmark mutation that leads to MEN1 syndrome [3-5]. Menin is essential during development and disruption of both *Men1* alleles in the mouse results in embryonic lethality thereby precluding analysis of its tumorigenesis potential [6]. Tissue-specific conditional inactivation of *Men1* in pancreatic islet β-cells, pituitary and in the parathyroid glands results in insulinomas, prolactinomas and parathyroid adenomas, respectively [7-9]. Taken together, these studies recapitulate the relevance of the *MEN1* locus in suppression of endocrine tumors.

Like inactivation of menin, loss of the retinoblastoma (RB) tumor suppressor protein promotes endocrine tumorigenesis. Mice heterozygous for *RB* are predisposed to develop pituitary tumors, although RB inactivation results in less frequent development of endocrine pancreas and parathyroid tumors [10]. Mice heterozygous for both *Men1* and *Rb1* alleles are predisposed to a high frequency of pancreatic hyperplasia and tumors of the intermediate pituitary suggestive of overlapping Menin and RB pathways in endocrine pancreas and pituitary tumorigenesis [11]. RB is a key regulator of the cell cycle and in its hypophosphorylated state restrains the E2F family of transcription factors that are required for DNA synthesis during the S-phase [12]. A cascade of cyclin-dependent kinases (Cdks) phosphorylates the eighteen serine/threonine residues of Rb and inactivates its tumor suppressor activity [13, 14]. Cdk activity is in turn inhibited by Cdk-inhibitors (CKI), such as the tumor suppressor p16Ink4a [15, 16]. p16INK4a, encoded by the *INK4A* locus, is a key inhibitor of Cdk4 and a central effector of cellular senescence pathways [17].

Previously, we and others showed that Cdk4 is indispensable for embryonic [18] and post-natal development [19] and regeneration [20] of endocrine pancreatic islet β-cells and proliferation of cells of the anterior pituitary [21, 22]. In contrast, inheritance of a p16^Ink4a^-insensitive *Cdk4*^Arg24Cys (R24C)^ allele, which results in an activated Cdk4^R24C^, increased the transformation potential of cells and predisposed mice harboring this mutation to cancer due to loss of RB tumor suppressor function [19, 23, 24]. Further, a recent study showed that Cdk4, not Cdk2, is required for tumorigenic proliferation in the pituitary and pancreatic islets [25]. In addition, p16^INK4a^ constrains islet cell proliferation and regeneration in an age-dependent manner [26], thus supporting the role of its substrate Cdk4 in regulating islet β-cell proliferation.

Along with inactivation of the RB/p16^INK4a^ pathway, activation of the enzyme telomerase is required to immortalize human epithelial cells [27]. In addition, loss of the three RB family members (RB, p107 and p130) leads to increased telomere length indicative of a role for RB family proteins in controlling telomere length [28]. Telomeres are specialized DNA elements that protect the ends of chromosomes from being recognized as DNA breaks from repair, recombination and degradation activities [29, 30]. However, ends of chromosomes continually lose the telomeres with each cell division due to incomplete replication of linear chromosomes by DNA polymerase. The ribonucleoprotein enzyme, telomerase, is responsible for adding new telomere repeats onto the 3′ ends of chromosomes. Telomerase has two essential components: an enzymatic telomerase reverse transcriptase catalytic subunit (TERT) and an RNA component (*TR or TERC*) that is used as a template in telomere synthesis. Most normal somatic cells possess low or undetectable telomerase activity, whereas, highly proliferative cells and majority of cancer cells possess elevated telomerase activity. Much of our present knowledge about the *in vivo* role of telomerase is derived from studies using the telomerase-deficient mouse model (*mTR*^−/−^ or *mTERC*^−/−^ mice) that was generated by elimination of the murine *TR* or *TERC* gene [31, 32] and the *TERT*^−/−^ mice [33-35]. Telomerase deficiency in combination with mutations in distinct tumor suppressor genes, except p53, significantly diminishes tumorigenesis potential [36-40] suggesting that telomerase inhibitors may be effective as anti-cancer agents [41-43]. Late-generation *mTERC*^−/−^ *Ink4a/Arf* mutant mice experienced a delayed tumor onset while maintaining the lymphoma and sarcoma spectrum [44]. In contrast, accelerated cancer onset and increased epithelial cancers were observed in late-generation *mTERC*^−/−^ *p53* mutant mice [37].

To date, the role of telomerase activity in the pathogenesis of multiple endocrine neoplasia is unclear and the potential utility of telomerase inhibitor therapy for these cancers has not been explored. The *Cdk4*^R24C^ mice are predisposed to increased incidence of endocrine pituitary tumors and, to a lesser extent, endocrine pancreatic adenomas [23, 24]. Here, using mice that harbor the *Cdk4*^R24C/R24C(R/R)^ mutation and inactivation of the *mTERC* locus, the *mTERC*^−/−^ *Cdk4*^R/R^ model, we have evaluated the consequence of RB pathway inactivation and telomere dysfunction on *in vivo* tumorigenesis. Due to their highly penetrant tumor development in the *Cdk4*^R/R^ model, we have especially focused on development of neoplasia of the endocrine pituitary and pancreas. We demonstrate that telomere dysfunction reduces the incidence of endocrine anterior pituitary and islet pancreas tumors in the *mTERC*^−/−^ *Cdk4*^R/R^ model. These results attest to an important role of telomerase activity in multiple endocrine neoplasias and support the utility of anti-telomerase inhibitors to target these tumors.

## RESULTS

### Tumor progression in Cdk4^R24C^ endocrine pancreas

**Figure 1:**
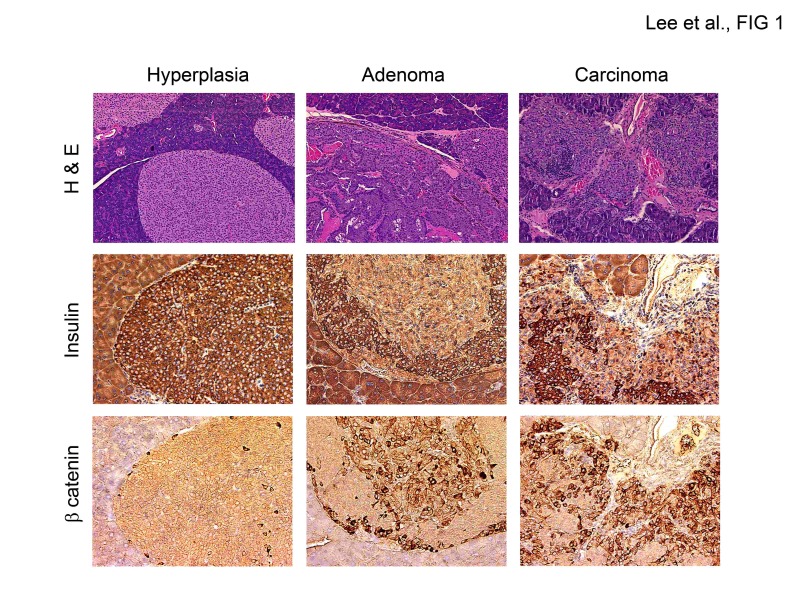
Tumor progression in Cdk4^R/R^ pancreas H&E staining and immunostaining (brown) for insulin and β-catenin in different pancreatic tumor stages from 12-month old Cdk4^R/R^ mice

Cdk4 is essential for the post-natal development of pancreatic islet β-cells and proliferation of the anterior pituitary [19, 21, 22]. In contrast, the *Cdk4*^R24C/R24C^ mice (*Cdk4*^R/R^ mice) are susceptible to increased tumor development within the pituitary and the endocrine pancreas [23, 24]. To better stage the tumor progression, we performed histological analysis on pancreas sections from one-year old *Cdk4*^R/R^ mice. These analyses revealed a distinct tumor development process in the endocrine pancreas; viz. (i) islet hyperplasia, (ii) islet adenomas, and infrequently, (iii) islet carcinomas (Figure 1). Abnormal localization of β-catenin is closely associated with carcinoma, tumor invasion and metastasis and poor survival and β-catenin localization is deregulated in human endocrine tumors [45]. β-catenin is transmembrane localized in normal cells, whereas in cancer cells, cytoplasmic or nuclear translocation of β-catenin is observed. Immunohistochemistry experiments indicated that β-catenin is localized to the membrane in hyperplastic endocrine islets in the *Cdk4*^R/R^ pancreas (Figure 1), suggesting that majority of the cells at this stage were non-tumorigenic. In contrast, β-catenin was predominantly localized to the cytoplasm or nucleus in islet adenomas and rare islet carcinomas (Figure 1). Normal insulin immunoreactivity was observed in hyperplastic islets indicative of normal β-cell differentiation (Figure 1). In contrast, we observed a dramatic reduction of insulin staining in several regions within the islet adenomas and infiltrating islet carcinomas (Figure 1). Interestingly, we observed an inverse correlation between β-catenin localization and insulin expression. Normal insulin expressing cells within the hyperplastic islets exclusively contained membrane localized β-catenin. In contrast, the cytoplasmic or nuclear β-catenin observed in islet adenomas and carcinomas was accompanied with dramatic loss of insulin expression indicative of a tumorigenic transition.

### Elevated telomerase activity and increased telomere length in *Cdk4*^R24C^ cells

**Figure 2:**
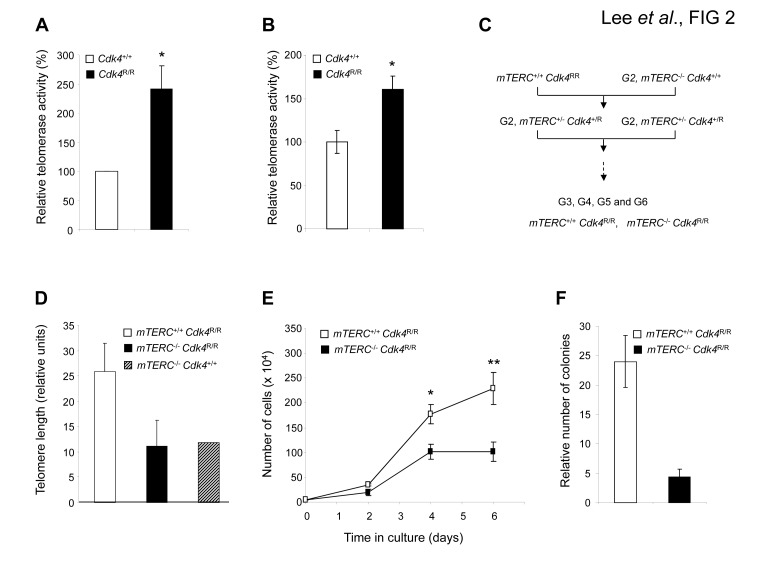
Role of telomerase activity in Cdk4^R/R^ mediated cell proliferation and transformation Telomerase Activity in islets (A) and MEFs (B) from Cdk4^+/+^ (open boxes) and Cdk4R/R (closed boxes) mice. TRAP assay was performed using 500 ng of protein from islets of 17 month old mice and from MEF cell lysate. (C) Mating scheme to generate mTERC^−/−^ Cdk4R/R and mTERC^+/+^ Cdk4R/R mice. Mating of Cdk4R/R mice with G2 mTERC^−/−^ mice produced heterozygous mTERC^−/+^ Cdk4^+/R^ mice and subsequently generationally aged mTERC^−/−^ Cdk4R/R and mTERC^+/+^ Cdk4R/R mice. (D) Average telomere length (in relative fluorescent units) of metaphase from MEF cultures from mTERC^+/+^ Cdk4R/R (open box), mTERC^−/−^ Cdk4R/R (closed box) and mTERC^−/−^ Cdk4^+/+^ (hashed box) mice as determined by Q-FISH. (E) Proliferation curves of mTERC^−/−^ Cdk4R/R (closed boxes) and mTERC^+/+^ Cdk4R/R (open boxes) MEFs. (F) Colony formation following seeding at 3500 cells of mTERC^−/−^ Cdk4R/R (closed boxes) and mTERC^+/+^ Cdk4R/R (open boxes) MEFs per 6 well plate and 14 days of culture. All data presented in experiments shown in each figure panel here represents the mean of at least three independent experiments and along standard error of mean. Statistical analysis was performed by student t-test.

Telomerase activation is correlated with the ability of cells to escape senescence, undergo immortalization and be susceptible to transformation [46]. We examined whether telomerase activity played a role in the *Cdk4*^R/R^ islet tumorigenesis model. Pancreas were harvested from old (>12 months of age) *Cdk4*^+/+^ and *Cdk4*^R/R^ mice and islets were isolated for analysis of telomerase activity. We observed a significantly elevated telomerase activity in *Cdk4*^R/R^ islets compared to islets isolated from comparatively old-aged *Cdk4*^+/+^ mice (Figure 2A) suggestive of a role for telomerase in islet tumorigenesis.

To better characterize the role of telomerase in the *Cdk4*^R/R^ model we performed studies with *Cdk4*^R/R^ mouse embryonic fibroblasts (MEFs). We and others previously showed that *Cdk4*^R/R^ MEFs escape cellular senescence, get immortalized upon repeated passage in culture and are susceptible to oncogenic transformation [23, 24]. To determine the role of telomerase in their transformation potential, we monitored telomere length and telomerase activity in *Cdk4*^R/R^ MEFs. We detected a significantly elevated telomerase activity in *Cdk4*^R/R^ MEFs compared to that observed in *Cdk4*^+/+^ MEFs (Figure 2B). Further, telomere based fluorescence *in situ* hybridization (FISH) analysis revealed an increased telomere length in *Cdk4*^R/R^ MEFs compared to that observed in *Cdk4*^+/+^ MEFs (data not shown).

### Generation of *mTERC* null and *Cdk4*^R24C/R24C^ mutant mice (*mTERC*^−/−^ *Cdk4*^R/R^ mice)

The elevated telomerase activity in *Cdk4*^R/R^ MEFs and islets are consistent with an important role for telomere biology in *Cdk4*^R24C^ driven cellular transformation and *in vivo* tumorigenesis. In order to directly examine the contribution of telomerase in this tumor model, we generated mice that (i) harbor the *Cdk4*^R24C^ mutation, and, (ii) that are null for the essential RNA component of mouse telomerase (*mTERC*^−/−^ *Cdk4*^R/R^ mice). To achieve this, we mated second generation, G2, *mTERC*^−/−^ mice [31] with *Cdk4*^R/R^ mice [19] to first derive heterozygous *mTERC*^+/−^ *Cdk4*^+/R^ mice that were subsequently inter-bred to produce G2, G3, G4, G5 and G6 *mTERC*^−/−^ *Cdk4*^R/R^ and *mTERC*^+/+^ *Cdk4*^R/R^ mice (Figure 2C). Diminished fertility precluded the derivation of subsequent generations of *mTERC*^−/−^ *Cdk4*^R/R^ and *mTERC*^+/+^ *Cdk4*^R/R^ mice. All experiments outlined in this manuscript have been performed on age and sex matched G5-G6, *mTERC*^−/−^ *Cdk4*^R/R^ and *mTERC*^+/+^ *Cdk4*^R/R^ mice.

### Growth characteristics of *mTERC*^−/−^ *Cdk4*^R/R^ mouse embryo fibroblasts

To evaluate the contribution of telomere dysfunction on *Cdk4*^R24C^ driven immortalization capacity and transformation potential, we generated MEFs from G5 *mTERC*^−/−^*Cdk4*^R/R^ and *mTERC*^+/+^ *Cdk4*^R/R^ mice. Quantitative fluorescence *in situ* hybridization (Q-FISH) on MEFs from *mTERC*^−/−^ *Cdk4*^R/R^ showed evidence of decreased telomere length compared to *mTERC*^+/+^ *Cdk4*^R/R^ MEFs (*p*=0.08, Figure 2D). As expected, the telomere length in *mTERC*^−/−^ *Cdk4*^R/R^ MEFs was similar to the telomere length observed in *mTERC*^−/−^ *Cdk4*^+/+^ MEFs (Figure 2D). These results indicate that the telomere length in *Cdk4*^R/R^ MEFs was most likely dependent on the elevated telomerase activity (Fig. 1B).

We next evaluated the cell growth properties and transformation potential of *Cdk4*^R/R^ MEFs in the context of telomere dysfunction. *Cdk4*^R/R^ cells can escape senescence and can be easily immortalized in culture [23]. All the assayed *mTERC*^−/−^ *Cdk4*^R/R^ and *mTERC*^+/+^ *Cdk4*^R/R^ MEF cultures were able to escape senescence upon repeat passage on a 3T3 protocol (data not shown). When assayed for their ability to proliferate in a continuous culture, we observed that *mTERC*^−/−^ *Cdk4*^R/R^ cells exhibited a reduced growth rate compared to *mTERC*^+/+^ *Cdk4*^R/R^ cells (Figure 2E). Actively transformed cells become growth factor independent that allows them to survive effectively in a low density seeded culture and proliferate efficiently to form colonies of cells. In contrast, non-transformed cells or cells with reduced transformation potential will remain growth arrested or maintain a reduced rate of proliferation in a low density culture and fail to form colonies. To evaluate their ability to proliferate as single cells beyond the senescence checkpoint and to compare their relative transformation potential, we next examined the ability of the *mTERC*^+/+^ *Cdk4*^R/R^ and *mTERC*^−/−^ *Cdk4*^R/R^ MEFs to grow in a low density culture (Figure 2F). Significantly reduced numbers of colonies were observed when the *mTERC*^−/−^ *Cdk4*^R/R^ MEF cells (4.3 ± 1.4 colonies; p<0.001) were grown in a low density culture compared to *mTERC*^+/+^ *Cdk4*^R/R^ MEFs (24.0 ± 4.4 colonies). These results are suggestive of reduced transformation potential of *mTERC*^−/−^ *Cdk4*^R/R^ MEF cells.

**Figure 3:**
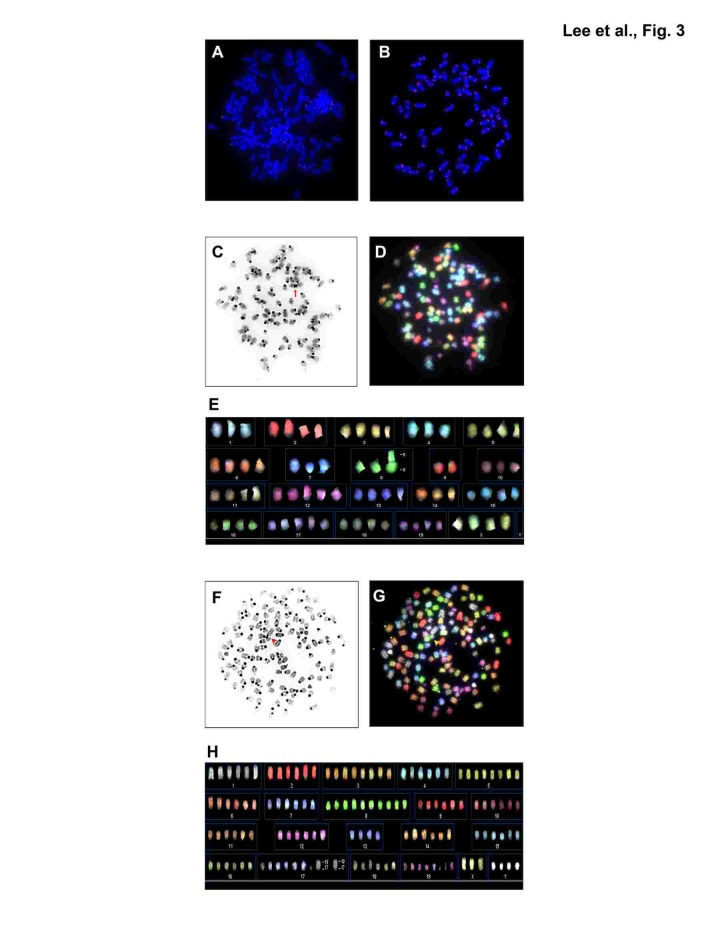
Representative telomere fluorescence of metaphase chromosomes from mTERC^−/−^ Cdk4^R/R^ (A) and mTERC^+/+^ Cdk4^R/R^ (B) MEFs Chromosomes were stained with DAPI (blue), and telomeric DNA was detected by FISH with Cy3-conjugated TTAGGG nucleic acid probe (red). Spectral karyotyping (D, E and G, H) shows chromosomal fusion and aneuploidy. Reverse DAPI image of the same metaphase as in D and G are shown in C and F. Arrow in C indicates Rb-like fusion. Arrows in F and G show examples of multi-radial chromosomes. Panels shown in C,D,E represent mTERC^−/−^ Cdk4^R/R^ and those in F,G,H represent mTERC^+/+^ Cdk4^R/R^.

Next, we performed cytogenetic analyses of *mTERC*^−/−^ *Cdk4*^R/R^ and *mTERC*^+/+^ *Cdk4*^R/R^ MEFs to determine the effect of telomere dysfunction on chromosomal aberrations in *Cdk4*^R/R^ cells (Table 1). The average chromosomal number for a major ploidy was 2n in *mTERC*^−/−^ *Cdk4*^+/+^ and *mTERC*^+/+^ *Cdk4*^+/+^ cells suggesting that telomere dysfunction does not result in aneuploidy (data not shown). In contrast, inheritance of the *Cdk4*^R24C^ mutation in *mTERC*^+/+^ *Cdk4*^R/R^ and *mTERC*^−/−^ *Cdk4*^R/R^ cells resulted in 4n or 6n as the average chromosomal number for a major ploidy. In addition, majority of *mTERC*^+/+^ *Cdk4*^R/R^ and *mTERC*^−/−^ *Cdk4*^R/R^ cells presented a high percentage of metaphases (between 33-47%) with other ploidy (4n or 6n). Together, these results are illustrative of *Cdk4*^R24C^ mediated increase in aneuploidy. Additionally, the *Cdk4*^R24C^ mutation leads to infrequent chromosomal rearrangements referred to as "sticky ends" that include three types of events: end-end associations, p-p or q-q or p-q fusions and Rb-like fusion (Table 1, Figure 3). We observed that inheritance of the *mTERC*^−/−^ background, in the *mTERC*^−/−^ *Cdk4*^R/R^ cells, increased the frequency of "sticky ends". In addition, we found that the number of nonreciprocal translocations were also higher in *mTERC*^−/−^ *Cdk4*^R/R^ cells (0.54 ± 0.43 per metaphase) compared to 0.09 ± 0.04 per metaphase in *mTERC*^+/+^ *Cdk4*^R/R^ cells (Table 1). Expectedly, the frequency of Robertsonian fusions - a hallmark of telomere dysfunction - increased to 95.0% ± 6.1 in *mTERC*^−/−^ *Cdk4*^R/R^ cells from 67.5% ± 16.7 in *mTERC*^+/+^ *Cdk4*^R/R^ cells (Table 1). Further, the frequency of chromosomes involved in tri and multi-radial fusion increased in *mTERC*^−/−^ *Cdk4*^R/R^ cells (31.8% ± 17.3) compared to 8.6% ± 1.9 in *mTERC*^+/+^ *Cdk4*^R/R^ cells (Table 1). Taken together, we conclude that inheritance of the *Cdk4*^R24C^ mutation leads to aneuploidy and chromosomal aberrations that synergize with the cytogenetic abnormalities incurred due to telomere dysfunction.

**Table 1:** Cytogenetic profiles in *mTERC*^+/+^ *Cdk4*^RR^, *mTERC*^−/−^ *Cdk4*^RR^ and *mTERC*^−/−^*Cdk4*^+/+^ fibroblast cultures

Culture	Passage	Metaphases Examined (chromosomes)	Average chromo. no. (major ploidy)	Percent Metaphases (other ploidy)	Rb-like fusions (%metaphases)	Tri-multi radial chromo involved.	non-reciprocal translocation/ metaphase	Fragments (hsr,dm) /metaphase	Average telomere length (relative units)
*mTERC*^+/+^ *Cdk4*^RR^#1	24	13 (597)	40 (2n)	33 (4n)	33	4.4	0.10	0.10	12.3
*mTERC*^+/+^ *Cdk4*^RR^#2	26	24 (1323)	124 (6n)	43 (4n)	57	7.5	0.17	0.58	34.5
*mTERC*^+/+^ *Cdk4*^RR^#3	24	20 (868)	39 (2n)	47 (4n)	80	10.4	0	0.07	27.7
*mTERC*^+/+^ *Cdk4*^RR^#4	26	20 (989)	81 (4n)	33 (6n)	100	11.9	0.10	0.50	29.2
*mTERC*^−/−^ *Cdk4*^RR^#1	23	17 (865)	77 (4n)	44 (6n)	100	19.3	0.22	0	10.7
*mTERC*^−/−^ *Cdk4*^RR^#2	23	20 (852)	74 (4n)	35 (6n)	100	>60	1.25	0	18.6
*mTERC*^−/−^ *Cdk4*^RR^ #3	23	20 (885)	77 (4n)	15 (2n)	85	16.2	0.15	0	4.4
*mTERC*^−/−^ *Cdk4*^+/+^	24	24 (329)	41 (2n)	8 (4n)	100	2.7	0	0.25	11.8

### Reduced telomere length and proliferative capacity in *mTERC*^−/−^ *Cdk4*^R/R^ mouse islet

**Figure 4:**
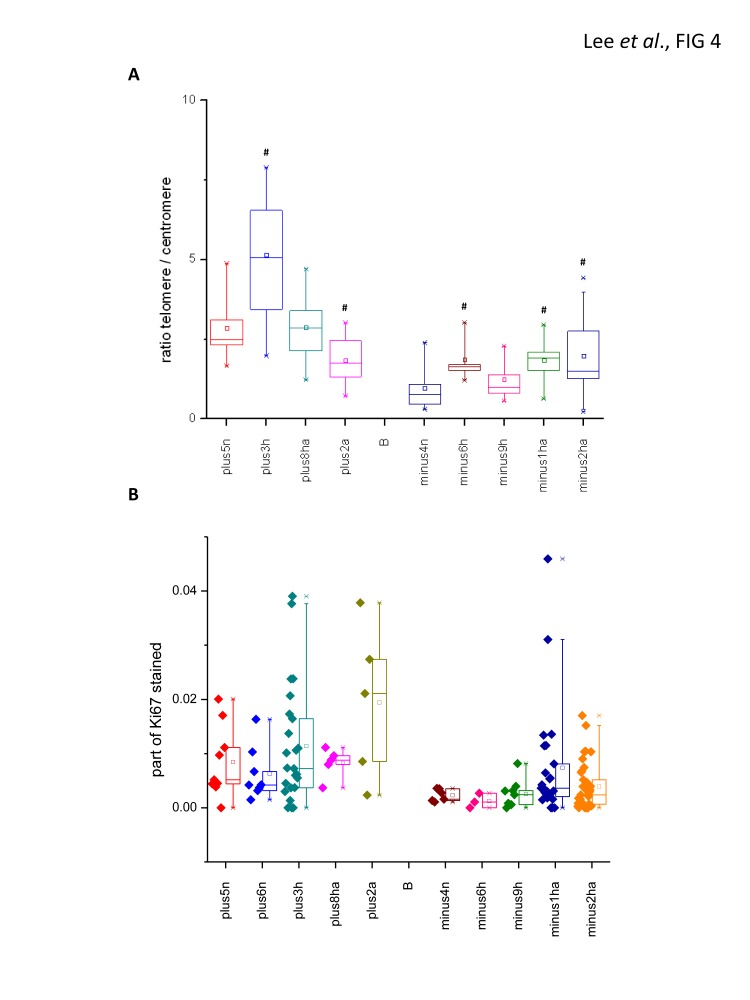
Telomere length and proliferation in islets from mTERC^−/−^ Cdk4R/R and mTERC^+/+^ Cdk4^R/R^ mice (A) Telomere length was analyzed in pancreatic islets from 4 telomerase positive and 5 negative mice. In each sample 8-22 islets were scored. In each islet, a ratio between telomere and centromere signal in phantom areas, which size was approximately equal to the size of cells was measured and the mean values is shown in the box chart plot. (B) And the same sample sections as for telomere length analysis were stained with Ki-67, insulin and DAPI antibodies. The total number of 8 m diameter circle-phantoms containing DAPI staining was calculated, which approximately corresponded to cell number (C). Then we estimated the number of the phantoms, which had Ki-67 staining (K). The part of stained cells was calculated as P=K/C and plotted on Y-axis for each islet. Statistical analysis for the ratio of telomere/centromere was done by ANOVA test. * indicates a value different from the normal islet in each group with a p < 0.05. Note: n=normal; h=hyperplasia; a=adenoma; plus= telomerase positive; minus= telomerase negative.

We next examined the telomere length and proliferation capacity in normal, hyperplastic and adenomatous islets from *mTERC*^+/+^ *Cdk4*^R/R^ and *mTERC*^−/−^ *Cdk4*^R/R^ mice to evaluate the role of telomere dysfunction on endocrine pancreatic tumorigenesis. The telomere/centromere ratios in the *mTERC*^+/+^ *Cdk4*^R/R^ mice were found to be higher than those observed in the *mTERC*^−/−^ *Cdk4*^R/R^ mice (Fig. 4A). In the *mTERC*^+/+^ *Cdk4*^R/R^ mice, the ratio of telomere/centromere in the hyperplastic sample (plus3h) is increased suggesting that telomeres are elongated, perhaps via telomerase activation. In contrast in the adenomatous sample (plus2a), the telomere/centromere ratio was reduced probably due to active proliferation (Fig. 4B), thus reducing telomere length. Further, this also suggests absence of telomerase-independent mechanisms in the *mTERC*^+/+^ *Cdk4*^R/R^ sample. Interestingly, the variation in centromere and telomere intensities between phantoms within one islet (both, in hyperplastic and adenomatous samples) was higher than that seen in the normal sample (plus5n). These observations suggest that in addition to telomere length, possibly the chromosome number varies in these samples. In the *mTERC*^−/−^ *Cdk4*^R/R^ samples, hyperplastic and especially hyperplastic/adenomatous samples showed increase of telomere/centromere ratio (Fig. 4A). These data suggest support the plausible existence of telomerase-independent mechanisms contributing to the increased telomere length (Fig. 4A) and increased cell proliferation (Fig. 4B) in the hyperplastic/adenomatous samples from the *mTERC*^−/−^ *Cdk4*^R/R^ mice.

### Telomere dysfunction suppresses *Cdk4*^R24C^ driven pituitary and endocrine pancreas neoplasia

**Figure 5:**
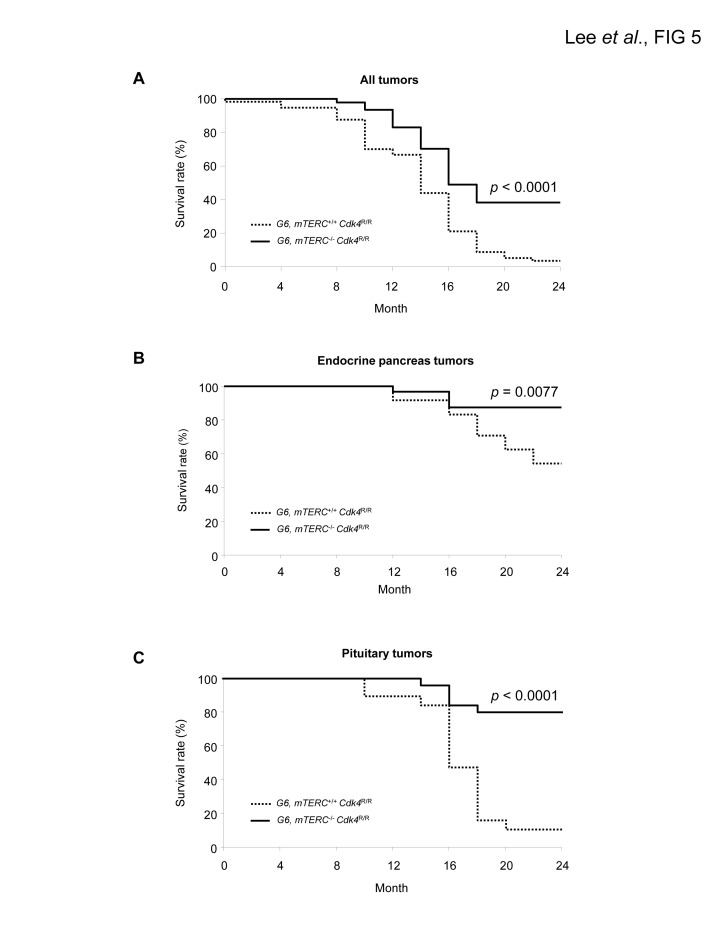
Kaplan-Meier analysis of tumor incidence in late generation mTERC^−/−^ Cdk4^R/R^ and mTERC^+/+^ Cdk4^R/R^ mice (A) Spontaneous tumors were diagnosed by macroscopic and microscopic observation for total tumor incidence. 47 mTERC^−/−^ Cdk4^R/R^ mice and 57 mTERC^+/+^ Cdk4^R/R^ mice were analyzed for total spontaneous tumor incidence. Mice with multiple tumors were counted once. Telomere dysfunction suppresses the incidence and delays the onset of endocrine pancreatic tumors (B) and pituitary tumors (C) in mTERC^−/−^ Cdk4^R/R^ mice. Pancreatic and pituitary tumors were diagnosed histologically. mTERC^−/−^ Cdk4^R/R^ (n=32) and mTERC^+/+^ Cdk4^R/R^ (n=27) mice were analyzed for pancreatic tumors and mTERC^−/−^ Cdk4^R/R^ (n=25) s from mT mTERC^+/+^ Cdk4^R/R^ (n=19) mice were analyzed for pituitary tumors. Statistical analysis was done by the log-rank test using SAS 9.1 program (SAS Institute Inc., Cary,NC).

Mice inheriting the *Cdk4*^R24C^ allele exhibit high rates of pituitary tumors and endocrine pancreas adenomas and, in addition, are susceptible to tumor development in other organs, specifically hemangiosarcomas [23, 24]. In order to appreciate the role of telomere dysfunction on *Cdk4*^R24C^ driven tumorigenesis, we characterized development of spontaneous tumors in G5-G6, *mTERC*^−/−^ *Cdk4*^R/R^ and G5-G6, *mTERC*^+/+^ *Cdk4*^R/R^ mice (hereafter referred to as *mTERC*^−/−^ *Cdk4*^R/R^ and *mTERC*^+/+^ *Cdk4*^R/R^ mice). Cohorts of mice were evaluated for two years and subjected to histopathology analysis to determine the incidence and types of tumors. These analyses revealed that spontaneous neoplasms occurred in both groups of mice, *mTERC*^−/−^ *Cdk4*^R/R^ and *mTERC*^+/+^ *Cdk4*^R/R^. Interestingly, we observed significantly reduced tumor development in the *mTERC*^−/−^ *Cdk4*^R/R^ group (29 of 47 mice; 62%), compared to the high tumor incidence in the *mTERC*^+/+^ *Cdk4*^R/R^ group (55 of 57 mice; 97%). According to the log-rank test, we observed a significant difference between the two groups (*p* < 0.0001). Kaplan-Meier estimation analysis revealed that the reduction in tumor incidence was correlated with an increase in survival (Figure 5A).

A subset of mice in both groups, *mTERC*^+/+^ *Cdk4*^R/R^ (n=27) and *mTERC*^−/−^ *Cdk4*^R/R^ (n=32) was subjected to further comprehensive analysis. As shown in Table 2, these analyses revealed that the total number of primary neoplasms was reduced in *mTERC*^−/−^ *Cdk4*^R/R^ mice (31 neoplasms in 32 mice; 18/32, 56% mice with neoplasms) compared to *mTERC*^+/+^ *Cdk4*^R/R^ mice (63 neoplasms in 27 mice; 25/27, 93% mice with neoplasms). Further, as shown in Table 2, we observed a reduction in the multiplicity of tumors in *mTERC*^−/−^ *Cdk4*^R/R^ mice (8/32 mice; 25% mice) compared to that seen in the *mTERC*^+/+^ *Cdk4*^R/R^ mice (18/27; 67% mice). Interestingly, the average age for tumor development was increased in *mTERC*^−/−^ *Cdk4*^R/R^ mice (males, 368 days and females, 423 days) compared to *mTERC*^+/+^ *Cdk4*^R/R^ mice (males, 307 days and females, 408 days). Together, these observation are indicative of reduced and delayed tumor initiation in the *mTERC*^−/−^ *Cdk4*^R/R^ mice compared to *mTERC*^+/+^ *Cdk4*^R/R^ mice.

**Table 2:** Characterization of tumors in *mTERC Cdk4*^R24C^ mutant mice

	*mTERC*^+/+^ *Cdk4*^RR^			*mTERC*^−/−^ *Cdk4*^RR^		
	Male	Female	Total	Male	Female	Total
Number of animals	7	20	27	13	19	32
*Tumor initiation*						
Animals with tumors (% of total animals)	7	18	25 (93%)	7	11	18 (56%)
Primary tumors (tumor/animal)	20	43	63 (2.33)	12	19	31 (0.97)
Animals with multiple tumors (% of total animals)	6	12	18 (67%)	2	6	8 (25%)
*Tumor progression*						
Benign tumors (% of total tumors)	13	26	39 (62%)	9	11	20 (65%)
Non-metastatic malignant tumors (% of total tumors)	7	17	24 (38%)	3	8	11 (36%)
Metastatic malignant tumors (% of total tumors)	2	4	6 (10%)	0	3	3 (10%)

Despite a reduction in tumor incidence, the histological appearance (tumor grade and severity) of the *mTERC*^−/−^ *Cdk4*^R/R^ tumors analyzed was indistinguishable from that of the *mTERC*^+/+^ *Cdk4*^R/R^ tumors. Further, as seen in Table 2, once tumor initiation occurred, the proportion of benign and malignant tumors were similar in the *mTERC*^−/−^ *Cdk4*^R/R^ mice (20/31; 65% benign and 11/31; 36% malignant) and *mTERC*^+/+^ *Cdk4*^R/R^ mice (39/63; 62% benign and 24/63; 38% malignant). Also, the number of malignant neoplasms that were metastatic was similar in *mTERC*^−/−^ *Cdk4*^R/R^ mice (3 in 31 tumors; 10%) compared to *mTERC*^+/+^ *Cdk4*^R/R^ mice (6 in 63 tumors; 10%). These results are suggestive of similar progression of established tumors in the *mTERC*^−/−^ *Cdk4*^R/R^ and *mTERC*^+/+^ *Cdk4*^R/R^ mice.

While neoplasms occurred in a number of organs at low incidence, highest tumor incidence occurred in the pituitary and the endocrine pancreas in both groups (Table 3). In addition, hemangiosarcomas were diagnosed in a number of organs with a relatively high incidence. The incidences of endocrine pancreatic (6/32; 19%) and pituitary (5/25; 20%) neoplasms were significantly reduced in *mTERC*^−/−^ *Cdk4*^R/R^ mice compared to *mTERC*^+/+^ *Cdk4*^R/R^ mice (Table 4). The reduction in occurrence of endocrine pancreas and pituitary neoplasms in *mTERC*^−/−^ *Cdk4*^R/R^ mice is further accentuated when sexes are combined to quantify neoplasm incidence (Table 4). The relative frequency of endocrine pancreas tumorigenesis was assessed in both the groups of mice by Kaplan Meier estimation (Figure 5B). These analyses revealed a significant reduction in endocrine pancreatic tumor development in *mTERC*^−/−^ *Cdk4*^R/R^ mice (4 of 32 mice; 13%) compared to those observed in the *mTERC*^+/+^ *Cdk4*^R/R^ mice (11 of 24 mice; 46%). Again, according to the log-rank test we observed a significant difference between the two groups (*p* =0.0077). Similarly, the relative frequency of pituitary tumorigenesis was assessed in both the groups of mice by Kaplan Meier estimation (Figure 5C). During 24 months, 17 of 27 *mTERC*^+/+^ *Cdk4*^R/R^ mice developed pituitary tumors and only 5 of 32 mice in *mTERC*^−/−^ *Cdk4*^R/R^ group developed pituitary tumors. According to the log-rank test, there was significant difference between two groups (*p* < 0.0001).

**Table 3:** Telomere dysfunction suppresses tumorigenesis in *Cdk4*^R24C^ mutant mice

Anatomic site, tumor type	*mTERC*^+/+^ *CdK4*^RR [a]^		*mTERC*^−/−^ *Cdk4*^RR [b]^	
	Incidence	Percentage	Incidence	Percentage
**Adrenal, cortical carcinoma**	0/27	0	3/32	9.4
**Adrenal, pheochromocytoma**	2/27	7.4	1/32	3.1
Hemangiosarcoma, various organs ^*^	11/27	41	4/32	12.5
**Lung, adenoma, alveolar**	1/27	3.7	2/32	6.3
**Lung, carcinoma, alveolar**	2/27	7.4	0/32	0
**Pancreas, islet cell adenoma + carcinoma**	16/27	59	6/32	19
**Pituitary, adenoma + carcinoma**	17/19	89	5/25	20
**Testis, interstitial cell tumor**	3/7	43	1/13	7.7

^*^ Liver, spleen, skin, mesenteric lymph node, heart, skeletal muscle, uterus.

^a^ Other neoplasma (n=1) in females are Harderian gland adenoma, Follicular center cell lymphoma, mammary glandular carcinoma, vertebral osteosarcoma and cutaneous sarcoma (n=2).

^b^ Other neoplasms (n=1) in females are histiocytic sarcoma, cecum adenoma and cutaneous sarcoma, in males, thymoma.

**Table 4:** Pancreatic and pituitary tumors in *mTERC Cdk4*^R24C^ mutant mice

	*mTERC*^+/+^ *Cdk4*^RR^			*mTERC*^−/−^ *Cdk4*^RR^		
	Male	Female	Total	Male	Female	Total
*Pancreas*						
Tissues examined	7	20	27	13	19	32
Adenoma, Islet cell (% of tissues examined)	4	11	15 (56%)	3	3	6 (19%)
Carcinoma, Islet cell (% of tissues examined)	0	1	1 (4%)	0	0	0 (0%)
Tumor incidence (% of tissues examined)	4	12	16 (59%)	3	3	6 (19%)
*Pituitary*						
Tissues examined	5	14	19	12	13	25
Adenoma, pars distalis (% of tissues examined)	0	5	5 (26%)	0	3	3 (12%)
Adenoma, pars intermedia (% of tissues examined)	3	6	9 (47%)	1	1	2 (8%)
Adenoma, NOS (% of tissues examined)	1	1	2 (11%)	0	0	0 (0%)
Carcinoma (% of tissues examined)	0	1	1 (5%)	0	0	0 (0%)
Tumor incidence (% of tissues examined)	4	13	17 (90%)	1	4	5 (20%)

## DISCUSSION

Intermediary proteins in the RB tumor suppressor pathway, Cdk4 and p16^Ink4a^, govern critical checkpoints that monitor cellular aging, senescence, immortalization capacity and transformation potential [13, 47, 48]. Both RB/p16Ink4a inactivation and telomerase activity are required to immortalize human epithelial cells [27]. However, mutation in the p53 tumor suppressor pathway appears to be obligatory for telomere-dysfunction induced tumor progression [37]. p16^Ink4a^ is encoded by the *INK4A* locus that also codes for the p19^ARF^ protein which regulates the p53 tumor suppressor pathway. Germline inactivation of the *INK4A* locus, which abrogates the function of both p16^Ink4a^ and p19^ARF^ [49], promotes tumorigenesis [50]. In conjunction with telomere dysfunction, inactivation of the *INK4A* locus results in suppression of cell transformation [38] and reduction of *in vivo* tumorigenesis [44]. Further, it was recently shown that *p16^INK4a^* exerts protective functions in proliferative cells bearing dysfunctional telomeres [51]. Here, using a mouse model that harbors a p16^Ink4a^-insensitive *Cdk4*^R24C^ mutation in concert with telomere dysfunction, we have examined the effects of Cdk4 and p16^Ink4a^ activities in telomere dysfunction regulated tumorigenesis. Further, the *Cdk4*^R24C^ model is highly susceptible to spontaneous tumors within the pituitary and endocrine pancreas making this model appropriate to study the tumor biology of multiple endocrine organs, a hallmark feature of MEN syndromes.

We demonstrate that cells with the *Cdk4*^R24C^ mutation display increased telomerase activity and telomere length. The *Cdk4*^R24C^ mutation co-operates with telomere dysfunction to enhance aneuploidy and chromosomal aberrations. However, telomere dysfunction reduces the cell growth rate and transformation potential of *mTERC*^−/−^ *Cdk4*^R/R^ cells. Further, *mTERC*^−/−^ *Cdk4*^R/R^ mice exhibit reduced tumor susceptibility primarily due to suppression of tumor initiation and increased latency of tumor development. Furthermore, the islet proliferation capacity in *mTERC*^−/−^ *Cdk4*^R/R^ mice was reduced. Strikingly, pituitary and endocrine pancreas tumorigenesis was significantly suppressed in *mTERC*^−/−^ *Cdk4*^R/R^ mice. Taken together, the decreased tumorigenesis and increased survival in *mTERC*^−/−^ *Cdk4*^R/R^ mice suggest that the significant telomere shortening and loss of telomere function reduces cancer incidence in mice homozygous for the *Cdk4^R24C^* allele.

The *Cdk4^R24C^* mutation promotes cell proliferation and aneuploidy in addition to inducing cytogenetic abnormalities. It is plausible that enhanced proliferation triggers the apoptotic elimination of cells at risk for transformation and thereby reduces tumor initiation in the *mTERC*^−/−^ *Cdk4*^R/R^ mice. Further, telomere dysfunction activates the p53-dependent double-strand break DNA damage pathway [52]. When the p53 checkpoint is subverted by inactivation of the *p53* locus, telomere dysfunction promotes rampant genomic instability and aggressive tumorigenesis in late-generation *mTERC*^−/−^ *p*53 mutant mice [37]. In contrast, an intact p53 checkpoint suppresses telomere-dysfunction induced carcinogenesis in the *Ink4a*/*Arf* mutant mice [38]. Our results of reduced *in vivo* tumorigenesis in the late generation the *mTERC*^−/−^ *Cdk4*^R/R^ mutant mice are consistent with those of Khoo *et al* that report a decrease in tumor incidence and increases tumor latency in late-generation *mTERC*^−/−^ *Ink4a*/*Arf* mutant mice [44]. We consider it plausible that telomere dysfunction in *mTERC*^−/−^ *Cdk4*^R/R^ mice elicits a p53-dependent DNA damage response that reduces tumor initiation.

Recent studies have postulated the importance of p53-mediated senescence as a potential mechanism that precludes *in vivo* tumorigenesis in cells experiencing telomere dysfunction [52-55]. p16^Ink4a^ controls the cellular senescence checkpoint and expression levels of p16^Ink4a^ increase in an age-dependent manner [15]. However, our study as well as the one published by Khoo et al., [44] are consistent with different pathways governing, (i) cellular senescence, and, (ii) telomere-dysfunction induced senescence. It is plausible that the RB/p16^Ink4a^ pathway, whereas required for cellular senescence and aging, may be dispensable for telomere dysfunction induced senescence. Instead, the p53 pathway may serve as the primary checkpoint that promotes telomere-dysfunction induced senescence and precludes tumor development in the setting of telomere dysfunction. As an extension, we speculate that tumors arising in the *mTERC*^−/−^ *Cdk4*^R/R^ mice may have incurred p53 pathway mutations that abrogate p53-mediated telomere induced senescence. Alternatively, tumors that develop in the *mTERC*^−/−^ *Cdk4*^R/R^ mice may have activated alternate (ALT) pathways of telomere elongation and maintenance to promote tumorigenesis [56]. It was recently shown that reactivation of telomerase in the setting of telomere dysfunction in a prostate cancer-prone *Pten* and *p53* null mouse enables malignant progression [57].

Telomerase activation is known to be a common event in human cancer and considered to be a useful marker for malignancy [46]. We show that telomere dysfunction effectively suppresses endocrine tumor initiation and increases tumor latency. These results, taken together, support the application of anti-telomerase inhibitor approaches to specific human cancers that harbor RB pathway mutations. However, we rationalize that the anti-telomerase inhibitor approach should be restrictive to tumors that harbor an intact p53 checkpoint. Telomerase activity is elevated in late stage islet tumors in the rat-insulin promoter driven SV40 T antigen mouse model of islet carcinogenesis [58]. We show here that *Cdk4*^R24C^ induced islet carcinogenesis involves increased telomerase activity. Telomere dysfunction significantly reduces endocrine pancreas and pituitary tumors in the *Cdk4*^R24C^ mice which is consistent with an important role for telomerase in endocrine tumorigenesis. The MEN1 mouse models reproducibly elicit the MEN1 syndrome with regards to increased susceptibility of tumors within the endocrine pancreas, pituitary and parathyroid organs [6-9]. However, the role of telomerase activation and telomere length regulation within the context of MEN1 mutations is unknown. Moreover, although telomerase activity is increased in many human malignancies, the role for telomerase activity in the human MEN syndrome, and specifically in MEN1, is obscure. Our results are strongly suggestive of elevated telomerase activity and deregulated telomere length in human MEN tumors and, thereby, advocate the utility of anti-telomerase inhibitors to treat multiple endocrine malignancies.

## EXPERIMENTAL METHODS

### Telomerase activity

Islets were isolated from 17-month-old *Cdk4*^+/+^ and *Cdk4*^R/R^ mice by collagenase digestion (Liberase, Roche Diagnostics, IN) followed by centrifugation over a Histopaque gradient (Sigma, St. Louis, MO). Islets were handpicked under a stereo microscope and were used for protein analysis. MEFs from *Cdk4*^+/+^ and *Cdk4*^R/R^ mice were isolated from individual embryos at 13.5 days as previously described [19, 23]. Telomerase activity was determined using TRAPeze telomerase detection kit (Intergene, NY). The same amount of protein (500ng/assay) was used for the telomerase activity detection.

### Generation of *mTERC*^−/−^ *Cdk4*^R/R^ mice

*Cdk4*^R/R^ mice were bred with *mTERC*^−/−^ mice to generate heterozygous *mTERC*^+/−^ *Cdk4*^+/R24C^ mice. These mice were inter-crossed to obtain successive generations of G2, G3, G4, G5 and G6 *mTERC*^−/−^ *Cdk4*^R/R^ and *mTERC*^+/+^ *Cdk4*^R/R^ mice. All experiments described in this study involved the use of G5 or G6 generation of mice. All animals were maintained under a 12 hour day/12 hour night cycle. All animal experiments were conducted under guidelines and protocols approved by the Animal Care and Use Committee and animal care was provided in accordance with the procedures outlined in the Guide for Care and Use of Laboratory Animals (National Research Council; 2011; National Academy Press; Washington, D.C.).

### Growth curve analysis and colony formation assays

For growth curve estimation, 5×10^4^ MEF cells from G5 mice were plated on 6-well plates and media was changed every three days. To test for the ability of cells to form colonies when plated at low density, MEF cells were seeded in triplicate at 3500 cells per 6-well plates and cultured in media containing 10% FBS. After 2 weeks of culture, cells were washed with PBS, fixed in methanol, stained with Giemsa stain and the number of colonies was counted.

### Quantitative telomeric FISH and spectral karyotyping

Telomere length and spectral karyotyping determinations were performed on MEF cultures as described previously [43]. Metaphase chromosomes and FISH of telomeric sequences were performed with Cy3 labeled TTAGGG peptide nucleic acid probe. Spectral karyotyping of MEFs was done according to the manufacturer protocol. At least 12 metaphases were analyzed for each sample.

### Tumor incidence and histopathology analyses

Mice were observed for 24 months for appearance of detectable or palpable tumors. Mice were examined closely for evidence of ill health or overt tumor growth during weekly inspections of the mouse colony. Tumor-bearing mice were euthanized and comprehensive necropsies were performed. Tissues were fixed in 10% buffered neutral formalin, paraffin-embedded, sectioned at 5 microns and stained with hematoxylin and eosin. At the end of 24 months, the remaining mice were euthanized and grouped into tumor-prone or tumor free categories. Mice were euthanized if profoundly ill or if external tumors exceeded 2cm in diameter and scored as a death in Kaplan-Meier survive analysis. Only those animals with a histologically proven cancer, as determined by a veterinary pathologist in the group (Dr. Miriam Anver), were scored as a tumor incidence event in the tumor analysis.

### Immunohistochemistry

Immunohistochemistry was performed by routine methods as described previously [59]. Normal and tumor tissues were fixed by immersion in either 10% neutral buffered formalin or 95% EtOH/5% glacial acetic acid overnight, dehydrated through ethanol and embedded in paraffin. Blocks were sectioned at 5Φm and stained with hematoxylin and eosin or prepared for immunohistochemistry by mounting tissue sections on electrostatically charged slides (ProbeOn Plus, Fisher Scientific). Antibody sources are, insulin (guinea pig anti-insulin polyclonal antibody, DAKO Inc., Indiana) and β-catenin (BD Transduction Laboratories, Cat No. 610153, Mouse IgG1). Analysis of immunohistochemistry was done to determine the distinct localization pattern (nuclear, cytoplasmic and nucleocytoplasmic).

### Telomere length and Ki-67 staining

Telomere length was analyzed in pancreatic islets from 4 telomerase positive and 5 negative mice by using iCys (CompuCyte, MA) image cytometry as described previously [43]. In each sample 8-22 islets were scored. Islets were defined by insulin staining, with exception of sample plus2a, which scores represented different regions from 2 big tumors that were not having insulin staining. In each islet, a ratio between telomere and centromere signal in phantom areas, which size was approximately equal to the size of cells was measured and the mean values is shown in the box chart plot. And the same sample sections as before for telomere length analysis were stained with Ki-67, insulin antibodies, and DAPI. The pancreatic islets on these sections were contoured. Within each contour, the total number of 8Φm diameter circle-phantoms containing DAPI staining was calculated, which approximately corresponded to cell number (C). Then we estimated the number of the phantoms, which had Ki-67 staining (K). The part of stained cells was calculated as P=K/C and plotted on Y-axis for each islet.
